# Investigation of the Global Changes in Photosynthetic Electron Transport in *Hosta* Plants Grown Under Different Light Levels

**DOI:** 10.3390/ijms252312876

**Published:** 2024-11-29

**Authors:** Dong-Huan Liu, Wen-Tao Ji, Qing-Qing Zou, Han-Yu Wu, Tao Li, Wen-Bin Shi, Chuang-Dao Jiang

**Affiliations:** 1 Beijing Botanical Garden, Key Laboratory of National Forestry and Grassland Administration on Plant Ex Situ Conservation, Beijing 100093, China; ldh1166@163.com (D.-H.L.); shige0622@sina.com (W.-B.S.); 2 State Key Laboratory of Plant Diversity and Specialty Crops, China National Botanical Garden, Institute of Botany, Chinese Academy of Sciences, Beijing 100093, China; jwtstyle@163.com (W.-T.J.); zouqingqingzoe299@163.com (Q.-Q.Z.); hanyuwu@ibcas.ac.cn (H.-Y.W.); litao@sxau.edu.cn (T.L.); 3 College of Forestry, Shanxi Agricultural University, Taigu 030801, China; 4 University of Chinese Academy of Sciences, Beijing 100049, China

**Keywords:** chlorophyll *a* fluorescence induction kinetics, light intensity, photosynthetic acclimation, proteomics, *Hosta*

## Abstract

To reveal the global regulation of photosynthetic electron transport (PET) in shade plants, the changes in chlorophyll *a* fluorescence induction kinetics (CFI) curves and proteomics were investigated using *Hosta* varieties. There was a significant difference in CFI curves between *Hosta* ‘Fire Island’ and other varieties (such as *Hosta* ‘Sum and Substance’) grown under weak light. Weak light induced the appearance of the W phase of CFI curves in the two varieties, which was consistent with a clear decrease in the oxygen-evolving complex and a large upregulation of photosystem (PS) II proteins. In *Hosta* ‘Fire Island’, the O-J rise of the CFI curves increased faster under weak light than under appropriate light, and this was not only accompanied by a large upregulation of the PS II protein but also a great downregulation in cytochrome b_6_/f, plastocyanin, and PS I. Moreover, weak light resulted in a considerable increase in photosynthetic rate and Rubisco abundance in *Hosta* ‘Fire Island’, yet the non-photochemical quenching and abundance of CP24 declined significantly. By contrast, weak light had fewer effects on these parameters in *Hosta* ‘Sum and Substance’. Therefore, we suggest that the PET is mainly affected by the abundance of PS II, oxygen-evolving complex, cytochrome b_6_/f, plastocyanin, and PS I in *Hosta* plants grown under weak light; meanwhile, the improved photosynthetic capacity under weak light was mainly related to the enhancement of light energy absorption and capture of PS II as well as the increase in the abundance of Rubisco.

## 1. Introduction

Light is the driving force of photosynthesis and also an important environmental factor that affects plant growth and development. Numerous studies have shown that the number of photosystem (PS) II reaction centers and the PS II antennae size increase in plants under weak light compared with those under high light, whereas PS I decreases or exhibits little change under high light [1,2,3,4,5]. At the same time, weak light also leads to a decrease in the number of other electron transporters (such as Cytb_6_f and P_C_) as well as Rubisco [6,7,8,9]. These changes result in lower photosynthetic electron transport (PET) activity and photosynthetic capacity in plants grown under weak light [7,10,11,12]. Based on their preference for light intensity, plants can be classified into two groups: sun plants and shade plants [10]. Sun plants have been intensively studied because they are widely distributed, whereas shade plants grown under weak light, or even extremely weak light, have received less attention. Studies using sun plants as materials do not provide a comprehensive picture of the PET regulation and photoacclimation mechanisms in shade plants. Therefore, shade plants (especially special species) are of particular significance for improving our understanding of photoacclimation.

Chlorophyll *a* fluorescence induction (CFI) kinetics is an important tool for studying photosynthetic function (including light absorption, capture, electron transport, and thermal dissipation) in plants [13,14,15,16]. When plant green tissues are sufficiently dark-adapted, the electron transporters on the acceptor side of P680, such as Q_A_, Q_B_, and PQ, lose all electrons and are oxidized [13,17], and, at this time, P680 is in a fully open state, with minimal transient fluorescence when illuminated. After exposure, P680 is excited to form P*680, which induces charge separation, and the resulting electrons gradually reduce the Q_A_, Q_B_, and PQ pools. The appearance of the J phase is mainly because the rate of the electron transport from P680 to reach Q_A_ is faster than that of the Q_A_ exchange with the oxidized PQ pool at the Q_B_ site [13,18]. Conversely, the appearance of the I phase is related to PS I primary photochemistry [13,17], which is closely related to the rate of reoxidation of the PQ pool mediated by the PS I primary photochemical reaction [19]. The highest transient fluorescence yield is observed when P680 is in a completely close state (P point). Theoretically, the CFI kinetics mainly reflect the primary photochemical activity of PS II and the redox state of the PQ pool and do not fully reflect the potential effects of changes in the abundance of other photosynthetic electron carriers. Quantitative proteomics can quantify all the proteins expressed by a genome or all the proteins in a complex mixed system, thus reflecting all protein content changes [7,20,21]. Therefore, the combination of proteomic and CFI curves should more comprehensively and accurately reveal the global regulation of PET.

*Hosta* is an important genus of shade-tolerant medicinal plants. Our previous studies have shown that it grows best under medium light intensity, and there are great differences between varieties grown under weak light or extremely weak light [22,23]. This suggests that there should be large differences in the regulation of PET and photoacclimation among *Hosta* varieties under weak light. Therefore, *Hosta* varieties with different light intensity sensitivities were carefully investigated using CFI kinetics and the proteomic techniques herein. This study is of great significance for understanding the regulation of PET, photoacclimation, cultivation management, and the selection of shade-tolerant varieties.

## 2. Results

### 2.1. Effects of Growth Irradiance on Morphology and CFI Kinetics Among Hosta Varieties

**Figure 1:**
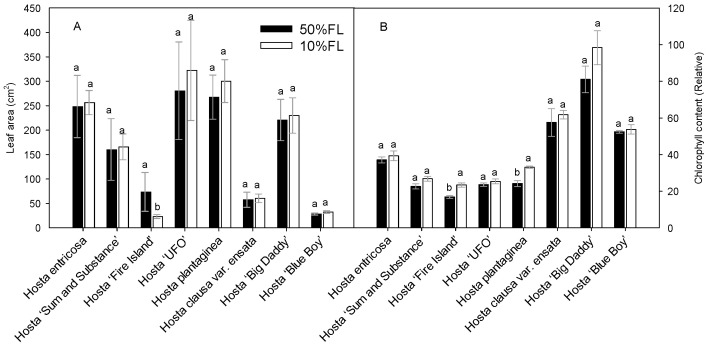
Changes in the leaf area (**A**) and chlorophyll content (**B**) of *Hosta* species and varieties grown under 50% (50%FL) and 10% full sunlight (10%FL). Different lowercase letters indicate significant differences between light treatments (*p* < 0.05 level).

First, the growth characteristics of *Hosta* species and varieties were compared under different light conditions (Figure 1). The results showed that leaf area varied considerably among the varieties, and their responses to the growth irradiances were also different. Compared with the appropriate growth irradiance [50% full sun light (FL)] treatment, most of the species and varieties showed an increase or slight increase in leaf area under weak light (10% FL), and only *Hosta* ‘Fire Island’ showed a significant decrease in leaf area (Figure 1). A 10% FL treatment resulted in an approximately 70% decrease in the leaf area of *Hosta* ‘Fire Island’ compared with 50% FL (Figure 1A). To further test this result, chlorophyll content was also measured. In all *Hosta* species and cultivars, the leaf chlorophyll content increased when grown at 10% FL (Figure 1B). Yet, only *Hosta* ‘Fire Island’ and *Hosta plantaginea* exhibited significant differences between the two light levels. These results suggest that *Hosta* ‘Fire Island’ may be more sensitive to weak growth light than other varieties.

**Figure 2:**
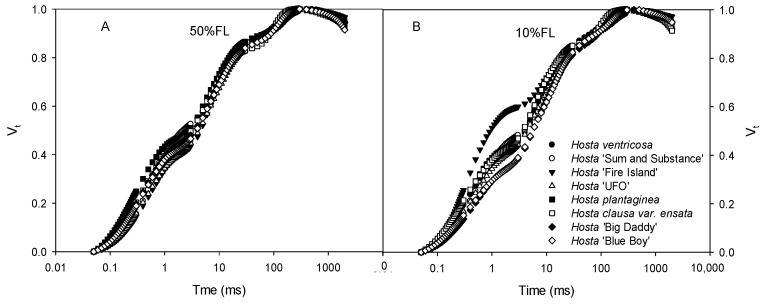
Changes in the chlorophyll *a* fluorescence induction (CFI) kinetics of *Hosta* species and varieties grown under 50% (50%FL, (**A**)) and 10% full sunlight (10%FL, (**B**)). The CFI curves are expressed as V_t_ = [(F_t_ − F_o_)/(F_P_ − F_o_)]. Each curve represents the averages of 20 independent measurements.

All *Hosta* species and varieties exhibited typical CFI curves (Figure 2). At 50% FL, all plants showed very slight differences in CFI curves. The fluorescence rise of *Hosta* ‘Fire Island’ was significantly accelerated under the 10% FL treatment (Figure 2), and the relative fluorescence yield at the J step (V_J_) was approximately 50% higher than that of the other species and varieties (Figure 2). This implies that there are large differences in PET between *Hosta* ‘Fire Island’ and other species and varieties.

### 2.2. Effects of Growth Irradiance on the Proteomes of Hosta ‘Fire Island’ and ‘Sum and Substance’

To further detect the differences in the characteristics of weak-light adaptation among *Hosta* species and varieties, proteomic analysis was performed with *Hosta* ‘Fire Island’ and ‘Sum and Substance’. There were significant differences in the protein abundance of various biological processes between the two varieties, many of which were related to photosynthesis. First, we observed that the 10% FL treatment resulted in an upregulation of the abundance of small subunits of Rubisco in ‘Sum and Substance’ and large subunits of Rubisco in ‘Fire Island’ (Table 1). We also noticed that PS II proteins were upregulated in both varieties under the 10% FL treatment, except that the magnitude of upregulation was higher in ‘Fire Island’ (Table 1). However, the abundance of the oxygen-evolving complex (OEC), an important component of the PS II complex, decreased in both varieties under weak light (Table 1). In contrast to the regulation of the abundance of the major PS II proteins, the 10% FL treatment resulted in a significant downregulation of the abundance of PS I and PS II antennae proteins. The extent of this downregulation was greater in *Hosta* ‘Fire Island’ than in *Hosta* ‘Sum and Substance’, and a greater number of associated protein species were also detected in the former (Table 1). Cytochrome b_6_/f (Cytb_6_/f) and plastocyanin (P_C_) are important electron transporters between the two photosystems [9]. The amounts of Cytb_6_/f and P_C_ were also downregulated in both varieties. Additionally, weak light resulted in a considerable decline in the abundance of CP24 in *Hosta* ‘Fire Island’, while this decline was not observed in *Hosta* ‘Sum and Substance’. The proteins in the leaves of *Hosta* ‘Fire Island’ showed greater downregulation in terms of variety and magnitude than those in the leaves of *Hosta* ‘Sum and Substance’ (Table 1).

### 2.3. Changes in CFI Curves Probed by Various Pulse Lights in Hosta ‘Fire Island’ and ‘Sum and Substance’

**Figure 3:**
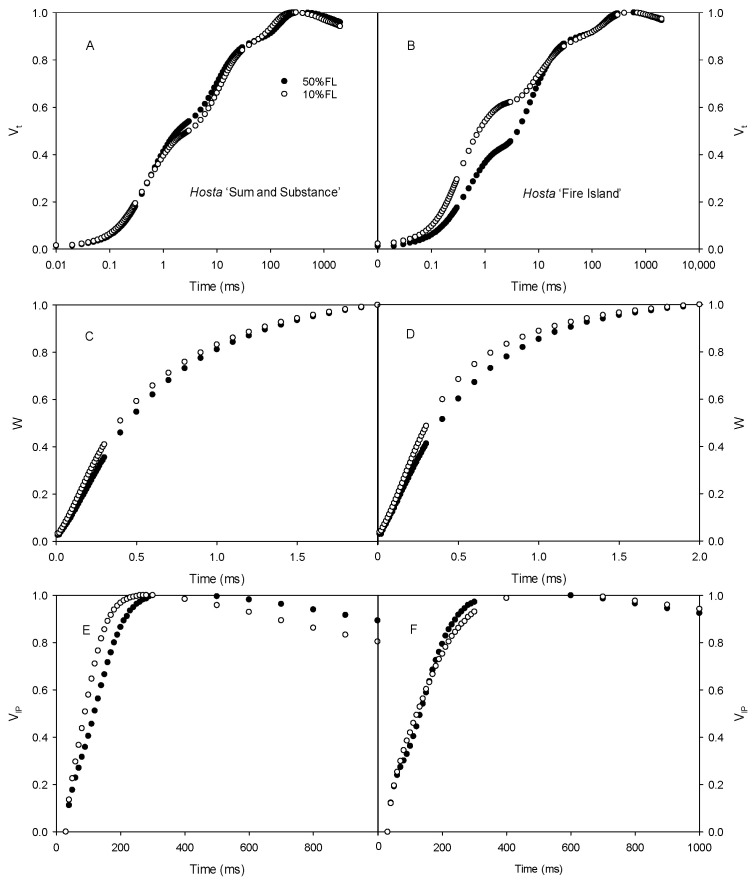
Changes in the chlorophyll *a* fluorescence induction (CFI) kinetics of *Hosta* ‘Sum and Substance’ (**A**,**C**,**E**) and ‘Fire Island’ (**B**,**D**,**F**) grown under 50% (50%FL) and 10% full sunlight (10%FL). (**A**,**B**): the CFI curves expressed as V_t_ = [(F_t_ − F_o_)/(F_P_ − F_o_)]; (**C**,**D**): the CFI curves expressed as W = [(F_t_ − F_o_)/(F_J_ − F_o_)]; (**E**,**F**): the CFI curves expressed as V_IP_ = [(F_t_ − F_I_)/(F_P_ − F_I_)]. The pulse light intensity in the CFI measurement was 3000 μmol m^−2^ s^−1^. Each curve represents the averages of 20 independent measurements.

First, the CFI kinetics were measured with saturated pulse light (3000 μmol m^−2^ s^−1^). As shown in Figure 3, the O-J fluorescence rise of *Hosta* ‘Fire Island’ accelerated significantly when grown under 10% FL compared with 50% FL, whereas it was slightly slowed down in *Hosta* ‘Sum and Substance’. This result demonstrates that PS II electron transport activity was reduced in the former and enhanced in the latter. In addition, the W phase of *Hosta* ‘Fire Island’ leaves significantly increased under 10% FL, while the difference in the I-P fluorescence rise was smaller compared with 50% FL (Figure 3). Interestingly, not only did the W phase of *Hosta* ‘Sum and Substance’ increase under 10% FL, but also the I-P fluorescence rise was significantly accelerated.

**Figure 4:**
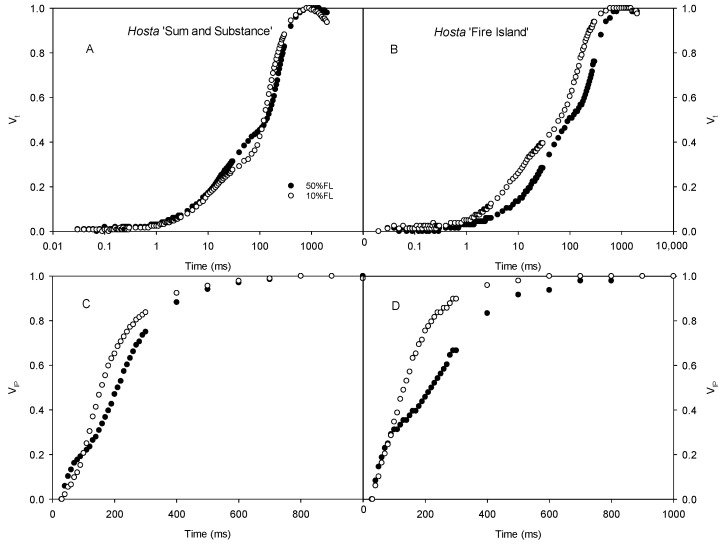
Changes in the chlorophyll *a* fluorescence induction (CFI) kinetics of *Hosta* ‘Sum and Substance’ (**A**,**C**) and ‘Fire Island’ (**B**,**D**) grown under 50% (50%FL) and 10% full sunlight (10%FL). (**A**,**B**): the CFI curves expressed as V_t_ = [(F_t_ − F_o_)/(F_P_ − F_o_)]; (**C**,**D**): the CFI curves expressed as V_IP_ = [(F_t_ − F_I_)/(F_P_ − F_I_)]. The pulse light intensity in the CFI measurement was 200 μmol m^−2^ s^−1^. Each curve represents the averages of 20 independent measurements.

We also determined the CFI curves under weak pulse light (200 μmol m^−2^ s^−1^) to further analyze the changes in the CFI curves. The results showed that there were clear differences in the O-J and I-P fluorescence rises of *Hosta* ‘Fire Island’ grown under 50% and 10% FL conditions. The difference in the O-J fluorescence rise of the CFI curve of *Hosta* ‘Sum and Substance’ was small under the two light treatments, whereas the I-P fluorescence rise was accelerated (Figure 4).

**Figure 5:**
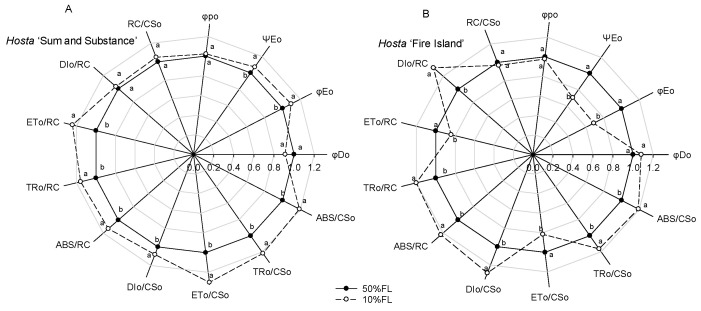
Changes in the JIP-test parameters of *Hosta* ‘Sum and Substance’ (**A**) and ‘Fire Island’ (**B**) grown under 50% (50%FL) and 10% full sunlight (10%FL). The JIP-test parameters were derived from Figure 3. Each value represents the average of 20 independent measurements. Different lowercase letters indicate significant differences between light treatments (*p* < 0.05 level).

Calculation of the CFI parameters indicated that there was little difference in the maximum quantum yield for primary photochemistry (φ_po_) in *Hosta* ‘Fire Island’ and ‘Sum and Substance’ when grown under the two light levels (Figure 5). Under the 10% FL treatment, the probability that a trapped exciton moves an electron into the electron transport chain beyond Q_A_^−^ (Ψ_Eo_) and the quantum yield for electron transport (φ_Eo_) increased more significantly in *Hosta* ‘Sum and Substance’, whereas these parameters in *Hosta* ‘Fire Island’ decreased substantially. Furthermore, 10% FL resulted in a decrease in the quantum yield of energy dissipation (φ_Do_) in *Hosta* ‘Sum and Substance’, whereas this value in *Hosta* ‘Fire Island’ slightly increased (Figure 5). Compared with the 50% FL treatment, the 10% FL treatment led to a significant increase in light energy absorption flux (ABS/CS_o_, ABS/RC), trapping flux (TR_o_/CS_o_, TR_o_/RC), and electron transport flux (ET_o_/CS_o_, ET_o_/RC) per cross-section and per reaction center in *Hosta* ‘Sum and Substance’, while the dissipation flux (DI_o_/CS_o_, DI_o_/RC) mildly increased (Figure 5). Under the 10% FL, although light energy absorption, capture, and dissipation flux per cross-section and per reaction center of *Hosta* ‘Fire Island’ were also substantially increased compared with the 50% FL, its electron transfer decreased significantly (Figure 5). The density of RCs (Q_A_-reducing PS II reaction centers) per cross-section (RC/CS_o_) was slightly increased in *Hosta* ‘Sum and Substance’, while the value of RC/CS_o_ was slightly decreased in *Hosta* ‘Fire Island’ (Figure 5). Accordingly, our results demonstrated that both the electron transport activities of PS I and PS II in *Hosta* ‘Sum and Substance’ and ‘Fire Island’ were influenced by growth irradiance.

### 2.4. Effects of Growth Irradiance on Photosynthetic Rate and Non-Photochemical Quenching in Hosta ‘Fire Island’ and ‘Sum and Substance’

**Figure 6:**
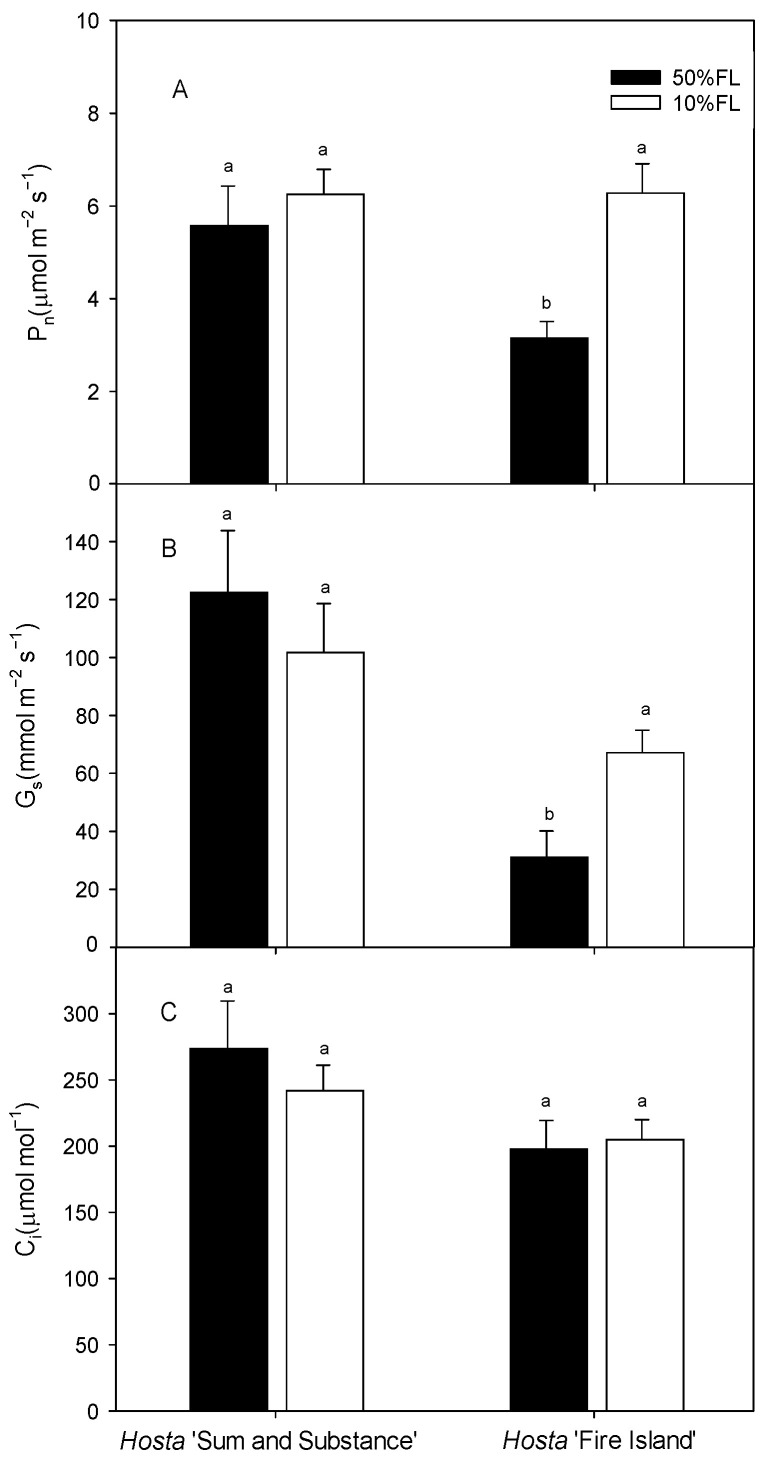
Changes in net photosynthetic rate (P_n_), stomatal conductance (G_s_), and intercellular carbon dioxide concentration (C_i_) of *Hosta* ‘Sum and Substance’ and ‘Fire Island’ grown under 50% (50%FL) and 10% full sunlight (10%FL). (**A**): net photosynthetic rate; (**B**): stomatal conductance; (**C**): intercellular carbon dioxide concentration. Each value represents the average of six independent measurements. Different lowercase letters indicate significant differences between light treatments (*p* < 0.05 level).

We then measured the photosynthetic rate by gas exchange. The results showed that the light-saturated photosynthesis rate (P_n_) of *Hosta* ‘Fire Island’ was approximately 3 μmol m^−2^ s^−1^ under the 50% FL treatment and increased to approximately 6 μmol m^−2^ s^−1^ under the 10% FL treatment. Additionally, the value of P_n_ in *Hosta* ‘Sum and Substance’ was approximately 5 μmol m^−2^ s^−1^ when it was grown under 50% FL, while it was slightly increased under 10% FL (Figure 6). The trends in stomatal conductance (G_s_) and intercellular carbon dioxide concentration (C_i_) were similar as those of P_n_ (Figure 6). All these data indicate there was little stomatal limitation with the improvement of photosynthesis in the two varieties under weak light.

**Figure 7:**
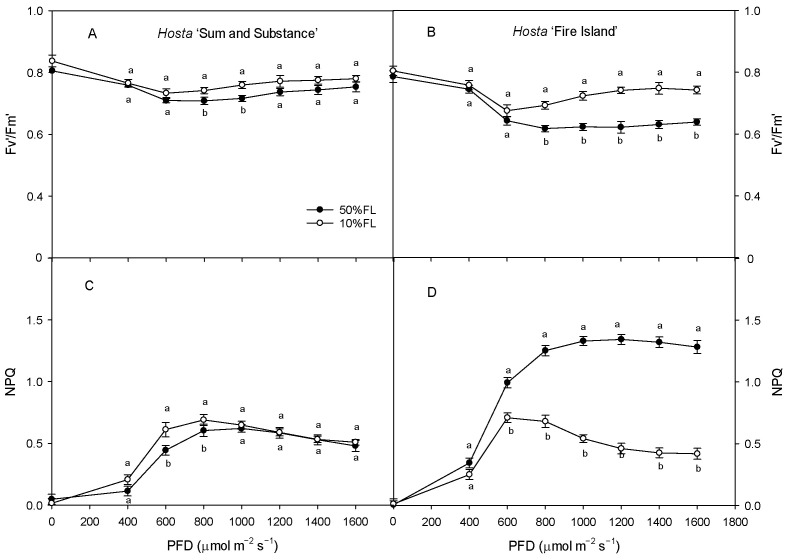
Changes in the efficiency of excitation energy capture by open PS II reaction centers (F_v_′/F_m_′) and non-photochemical quenching (NPQ) of *Hosta* ‘Sum and Substance’ and ‘Fire Island’ grown under 50% (50%FL) and 10% full sunlight (10%FL). (**A**,**B**): the efficiency of excitation energy capture by open PS II reaction centers; (**C**,**D**): non-photochemical quenching. Each value represents the average of six independent measurements. Different lowercase letters indicate significant differences between light treatments (*p* < 0.05 level).

With the increase in light intensity, the efficiency of excitation energy capture by open PS II reaction centers (F_v_′/F_m_′) tended to decrease in both varieties under different growth light intensities, while the non-photochemical quenching (NPQ) increased gradually, with both of these ultimately stabilizing (Figure 7). The value of F_v_′/F_m_′ in *Hosta* ‘Fire Island’ grown under the 10% FL treatment was significantly higher than that under the 50% FL treatment, while the level of NPQ was significantly lowered. Both these parameters in *Hosta* ‘Sum and Substance’ grown under the 10% FL treatment exhibited little change from those grown under the 50% FL treatment (Figure 7). Apparently, the regulation of excitation energy capture and non-photochemical quenching was greater in *Hosta* ‘Fire Island’ than in ‘Sum and Substance’.

## 3. Discussion

### 3.1. Generalities in the Regulation of PET and Photoacclimation of Hosta Varieties

In this study, all *Hosta* species and varieties were able to maintain growth under 10%FL treatment (Figure 1). The proteomics data showed that 10% FL induced a significant downregulation of the abundance of PS I antenna proteins in *Hosta* ‘Fire Island’ and ‘Sum and Substance’ (Table 1). By contrast, the CFI kinetics data showed that 10%FL treatment resulted in an increase in ABS/CS_o_ and TR_o_/CS_o_ in both varieties (Figure 5). We noticed that the content of chlorophyll tended to increase in both cultivars when grown at 10%FL (Figure 1B). Although the proteomic data showed a substantial downregulation of the amount of PS I antennae, there was no further substantial increase in the abundance of PS II antenna pigment proteins. This may be due to the fact that the number of PS II is much larger than that of PS I [3,9,10]. So, the extensive downregulation of PS I antenna proteins in *Hosta* is not necessarily accompanied by a significant increase in the amount of PS II antenna pigment proteins (Table 1). Most likely, the extensive downregulation of PS I antenna proteins in *Hosta* is a regulatory strategy to balance the light energy absorption and capture of PS II under weak light.

Regulation of PET plays an important role in the photoacclimation of plants to various growth irradiances [3,4,5,6,11]. In this study, significant upregulation of the major PS II proteins (such as D1) occurred in *Hosta* ‘Fire Island’ and ‘Sum and Substance’ grown under 10%FL (Table 1), indicating the number of PS II reaction centers increased markedly. Yet, we noticed that there was an obvious downregulation of the oxygen-evolving complexes (OEC). Moreover, the growth in 10%FL induced an increase in the W phase in both varieties (Figure 3). It was shown that the rate of electron transport from water to Yz was significantly lower than from Yz to the PS II reaction center, and thus the rate-limiting step was the OEC [13,15,24]. It is reported that either OEC injury or uncoupling results in a rapid increase in W phase fluorescence yield [13,24]. In the present study, the increase in the W phase was consistent with the downregulation of OEC protein abundance (Figure 3; Table 1). Therefore, the donor electron transfer activity of PS II in the two varieties may be suppressed to a certain extent. Most likely, the downregulation of the OEC complex protein abundance and the decrease in activity may result in a very slight increase in active PS II reaction centers (RC/CS_o_) in both varieties (Figure 6). The inactive PS II reaction centers owing to the absence of OEC in *Hosta* leaves may have existed as a specific dissipation mechanism, which is consistent with increased DI_o_/CS_o_ in ‘Fire Island’ and ‘Sum and Substance’. Due to the increased ABS/CS_o_ and TR_o_/CS_o_, the lower number of active PS II reaction centers may further contribute to the increased ABS/RC and TR_o_/RC (Figure 6).

In the present study, there was a significant downregulation of PS I and an associated protein enrichment in *Hosta* leaves when grown under 10%FL (Table 1). It has been shown that the relative change in V_IP_ reflects the rate of reoxidation of the PQ pool by PS I and is related to PS I activity [15,19]. The CFI kinetics analyses revealed that the increase in the I-P fluorescence rise was significantly accelerated under weak pulse light excitation both in *Hosta* ‘Fire Island’ and ‘Sum and Substance’ under 10%FL (Figure 5), and thus PS I-mediated electron transfer activity decreased to some extent. This also suggests that the downregulation of PS I abundance and activity is an important regulatory strategy for PET in the two varieties. Because PS I electron transfer is faster than that of PS II [15,17,19], the decrease in the PS I electron may help to match electron transfer between the two photosystems when *Hosta* is grown under weak light.

Rubisco is the key enzyme in the dark reaction of photosynthesis. It has been reported that a decrease in the amount of Rubisco under weak light leads to a decrease in photosynthetic rate in sun plants [6,7,8]. In this study, the 10%FL treatment resulted in an upregulation of the amount of Rubisco, which was consistent with the increasing trend of P_n_ in *Hosta* ‘Fire Island’ and ‘Sum and Substance’ (Figure 6; Table 1). Therefore, the maintenance of the higher photosynthetic capacity of both varieties under weak growth light should be related to the increase in the abundance of Rubisco.

In conclusion, weak growth light downregulated the function of PS I but improved light energy absorption and capture of PS II in *Hosta* ‘Fire Island’ and ‘Sum and Substance’. The higher photosynthetic capacity of both varieties under weak growth light was mainly related to the enhancement of the light energy absorption and capture of PS II as well as the increase in the abundance of Rubisco.

### 3.2. Differences in the Regulation of PET and Photoacclimation of Hosta Varieties

Although some common strategies were used by the *Hosta* varieties for adapting to weak growth light, some differences still existed. Our results showed that the leaf area of *Hosta* ‘Fire Island’ grown under 10%FL was significantly lower than that of plants grown under 50%FL, whereas none of the other species and varieties (including *Hosta* ‘Sum and Substance’) exhibited a significant difference in leaf area as a function of growth light intensity (Figure 1). In addition, there was a distinct increase in the chlorophyll content of *Hosta* ‘Fire Island’ under 10%FL. More importantly, 10%FL led to wide and significant decreases in PS I light-trapping pigment proteins in *Hosta* ‘Fire Island’, while fewer decreases occurred in *Hosta* ‘Sum and Substance’ (Table 1). These results demonstrated that the growth of *Hosta* ‘Fire Island’ was more sensitive to weak light.

In addition to the antennae pigments, the major proteins of the PS II reaction center of *Hosta* ‘Fire Island’ grown under 10%FL were widely and significantly upregulated relative to that of plants grown under 50%FL, whereas the cytb_6_f, P_C_, and PS I reaction center-related proteins were all significantly downregulated (Table 1). This means that there may be an increase in the number of electrons transferred from PS II to Q_A_ and a decrease in the number of electrons transferred afterwards (Q_A_). However, the related proteins in *Hosta* ‘Sum and Substance’ were regulated in fewer categories and to a lesser extent than those in *Hosta* ‘Fire Island’. The difference in the protein abundance implied that there was a large difference in the electron transport of both varieties. The 10%FL also resulted in a substantial increase in the O-J fluorescence rise of *Hosta* ‘Fire Island’, indicating a decrease in electron transfer from Q_A_ to Q_B_ on the PS II acceptor side. Further calculations of various fluorescence parameters revealed that the ET_o_/CS_o_ and Ψ_Eo_ of *Hosta* ‘Fire Island’ grown under 10%FL were lower than those under 50%FL conditions (Figure 5), demonstrating that its PS II electron transport was significantly suppressed by weak growth light. On the contrary, there was a more significant increase in the electron transport of PS II in *Hosta* ‘Sum and Substance’, as reflected by the increase in ET_o_/CS_o_ and Ψ_Eo_ (Figure 5). In the photosynthetic electron transfer chain, cytb_6_f and P_C_, which are located between PS II and PS I, may affect the electron transport on the acceptor side of PS II by influencing the oxidation of the PQ pool, which in turn may lead to an increase in the J phase. The amount of electron transfer between PS II and PS I in *Hosta* ‘Fire Island’ (including cytb_6_f and P_C_) was significantly reduced, especially P_C_ (Table 1). On the contrary, the lesser downregulation of cytb_6_f and P_C_ (especially P_C_) abundance in *Hosta* ‘Sum and Substance’ is consistent with its lack of a significant increase in the J phase. Therefore, these may reflect the main differences in the regulation of electron transport between *Hosta* ‘Fire Island’ and ‘Sum and Substance’ during their adaption to a weak-light environment; the occurrence of the J phase in this study may be mainly related to the large upregulation of the PS II protein and a significant reduction in the abundance of cytb_6_f and P_C_.

In addition, CFI measurements with a weak pulse light (200 μmol photons m^−2^ s^−1^) showed that 10%FL resulted in a significantly faster I-P rise of *Hosta* ‘Fire Island’ and ‘Sum and Substance’ (Figure 4). This suggests that at this time the PS I is the limiting site for electron transfer in both varieties. When measuring with a strong pulse light, the I-P fluorescence rise of *Hosta* ‘Sum and Substance’ grown under 10%FL still increased faster than that of the plants grown under 50%FL (Figure 3). However, the fluorescence difference between *Hosta* ‘Fire Island’ grown under the two light treatments was not significant (Figure 4), confirming that PS I more seriously affected the photosynthetic electron transfer of *Hosta* ‘Sum and Substance’. Therefore, we deduced that the electron transfer of PS II, cytb_6_f, and P_C_ in the leaves of *Hosta* ‘Fire Island’ was able to maintain an unimpeded state with PS I limiting the electron transfer when the amount of electron transfer was low under weak pulse light. Additionally, the limiting effects of cytb_6_f and P_C_ were enhanced when the amount of electron transfer increased at saturating pulse light, which somewhat mitigated or masked the limitation of PS I in *Hosta* ‘Fire Island’ plants. However, in *Hosta* ‘Sum and Substance’, PS I was the limiting site for electron transport due to the lack of abundance regulation by cytb_6_f and P_C_. From this perspective, the extensive, large decreases in PS I antennae and core proteins in *Hosta* ‘Fire Island’ may be an adaptation to the decline in electron transfer on the PS II receptor side.

Generally, weak growth light causes a decrease in photosynthetic rate in many sun plants [7,8,10,11]. However, as *Hosta* is a shade-tolerant plant, its photosynthetic rates increase under 10%FL (Figure 6). For *Hosta* ‘Sum and Substance’, there was a small increase in the abundance of Rubisco-related proteins and a slight increase in P_n_; however, the abundance of Rubisco was significantly upregulated in *Hosta* ‘Fire Island’, and there was a large increase in the P_n_ (Figure 6; Table 1). We noticed that the slight increase in the P_n_ of *Hosta* ‘Sum and Substance’ under 10%FL was accompanied by an increase in PS II electron transfer activity; however, the significant increase in the P_n_ of *Hosta* ‘Fire Island’ under 10%FL was accompanied by a clear decrease in PS II electron transfer activity (Figure 5 and Figure 6). Actually, *Hosta* ‘Fire Island’ had a strong heat dissipation capacity when grown at 50% FL, which was significantly reduced when grown at 10% FL (Figure 7). The decline in NPQ was consistent with the obvious deregulation in the abundance of CP24 (Figure 7, Tabel 1). Meanwhile, both the levels of NPQ and the abundance of CP24 were hardly changed in *Hosta* ‘Sum and Substance’. This suggests that the part of the transmembrane gradient formed through PET, which thus drives heat dissipation, would be considerably reduced in weak growth light, and the potion used for assimilation would be increased correspondingly. Therefore, this could lead to a decrease in the electron transport activity of *Hosta* ‘Fire Island’, while the photosynthetic rate is increased instead. However, the decrease in NPQ in *Hosta* ‘Fire Island’ is accompanied by a marked increase in DI_o_/CS_o_, at which point the PS II reaction center with lowered oxygen-evolving activity is likely to serve as an alternative heat dissipation mechanism (Figure 3 and Figure 6).

Therefore, *Hosta* ‘Sum and Substance’ adapts to weak growth light by improving electron transfer and photosynthetic capacity to a certain extent; for *Hosta* ‘Fire Island’, the significant downregulation of electron transfer, heat dissipation, and improved photosynthetic capacity are unique adaptation strategies under weak growth light.

## 4. Materials and Methods

### 4.1. Plant Materials and Experimental Design

The experiments were conducted at the Institute of Botany, Chinese Academy of Sciences, from May to September in 2020 and 2021. *Hosta* varieties (including *Hosta plantaginea*, *Hosta entricosa, Hosta* ‘UFO’, *Hosta clausa* var. *normalis*, *Hosta* ‘Sum and Substance’, *Hosta* ‘Blue Boy’, *Hosta* ‘Fire Island’, and *Hosta* ‘Big Daddy’) were planted in plastic pots (pot diameter: 29 cm and pot height: 30 cm). The substrate in the pots consisted of grass charcoal and soil (1:1, *v*/*v*). The *Hosta* plants were placed outdoors and divided into two groups: appropriate light (50% full sun light by shading) and weak light (10% full sun light by shading) treatments. On clear days, the daily maximum photosynthetically photon flux density was about 1500 μmol m^−2^ s^−1^ at noon in Beijing. Regular watering and fertilizing were performed throughout the experiment to avoid drought and nutrient stress. After 5 weeks of growth under the light treatment, all measurements were conducted using the just fully expanded leaves (the third leaf from the inside out).

### 4.2. Determination of Leaf Area and Chlorophyll Content

The leaf area (the third leaf from the inside out) of Hosta was determined with a leaf area meter (Li-3000A, Li-COR, Lincoln, NE, USA); meanwhile, the chlorophyll content was measured with a chlorophyll meter (SPAD-502 Plus; Konica Minolta, Inc., Japan). The determinations were repeated at least 20 times for each variety and treatment.

### 4.3. Determination of Gas Exchange

A portable gas exchange system (Ciras-2, PP Systems, Amesbury, MA, USA) was used to measure net photosynthetic rate (P_n_), stomatal conductance (G_s_), and intercellular carbon dioxide concentration (C_i_) between 8:00 am and 12:00 pm on a clear day in June and July. At least 6 leaves were determined for each light treatment of all varieties. The light intensity, CO_2_ concentration, and air humidity were maintained at 1200 ± 15 μmol m^−2^ s^−1^, 380 ± 20 μmol mol^−1^, and 80 ± 5%, respectively, with an ambient temperature kept in the leaf chamber.

### 4.4. Determinations of CFI Kinetics

Following the protocols of Strasser et al. [25] and Zou et al. [26], the CFI measurement was conducted with a Handy Plant Efficiency Analyzer (Handy-PEA, Hansatech Instruments, Narborough, UK) in vivo. The measurements were made after full dark adaptation (1 h) of the leaves at 8:00 pm. Pusle irradiation consisted of 1 s of red radiation (650 nm peak wavelength) provided by an array of four LEDs (light-emitting diodes) focused on the surface of a leaf with a diameter of 5 mm. The pulse light intensities for the experiment were 3000 and 200 μmol photons m^−2^ s^−1^, respectively. The first reliably measured point of the CFI was at 20 µs, which was used as the minimum fluorescence (F_o_). The following data were obtained: fluorescence intensity at 2 ms [J phase (F_J_)], fluorescence intensity at 30 ms [I phase (F_I_)], and maximum fluorescence intensity (F_P_). The relative variable fluorescence was calculated as follows: V_t_ = (F_t_ − F_o_)/(F_P_ − F_o_), where F_t_ is the measured fluorescence intensity at time t between F_o_ and F_P_; W = [(F_t_ − F_o_)/(F_J_ − F_o_)]; V_IP_ = [(F_t_ − F_I_)/(F_P_ − F_I_)]. Twenty independent measurements were made for each treatment. The JIP-test parameters were calculated according to the method of Strasser et al. [1,25].

### 4.5. Determination of Rapid Chlorophyll a Fluorescence Light Response Curve

Following our previous method [20], chlorophyll *a* fluorescence quenching was conducted in vivo at room temperature using a pulse-modulated fluorimeter (FMS-2, Hansatech, Narborough, UK). Before measurement, all plants were fully dark-adapted (for 1 h). The minimal fluorescence (F_o_) was determined with the measuring light, while the maximum fluorescence (F_m_) was examined after a saturating pulse (>8000 μmol m^−2^ s^−1^). The fluorescence measurement protocol was as follows: fully dark-adapted leaves were continuously illuminated by actinic light from the FMS-2 light source. The actinic light intensities were 0, 400, 600, 800, 1000, 1200, 1400, 1600, 1800, and 2000 μmol m^−2^ s^−1^. The steady-state fluorescence levels (F_s_) and the maximum chlorophyll yield in the light-adapted state (F_m_′) were recorded by applying successive saturating pulses. Once a steady state was reached, far-red light was applied for the determination of F_o_′ after the actinic light had been switched off. All fluorescence quenching parameters (including the efficiency of excitation energy capture by open PS II reaction centers (F_v_′/F_m_′) and non-photochemical quenching (NPQ)) were calculated according to the method of Demmig-Adams and Adams [27]. At least six replicates were tested for each treatment.

### 4.6. Proteomic Analysis

After 5 weeks of growth, the just fully expanded leaves (the third leaf from the inside out) were collected for proteomic analysis. Three independent samples under light treatments were utilized in this study. (1) Protein extraction and digestion were performed according to the filter-assisted sample preparation method described by Wisniewski et al. [20]. Briefly, frozen samples were transferred into low-protein-binding tubes (2 mL Eppendorf). The liquid nitrogen was added to each sample and ground thoroughly. The samples were then supplemented with an extraction buffer of 1 mL and mixed, and the mixtures were added with Tris-phenol buffer and mixed for 30 min at 4 °C. Furthermore, the mixtures were centrifuged at 7100× *g* for 10 min at 4 °C to collect phenol supernatants. The volume of a 0.1M cold ammonium acetate-methanol buffer was added 5 times to the supernatants and precipitated at −20 °C overnight. After precipitation, the samples were centrifuged at 12,000× *g* for 10 min to collect the precipitation. Then, the precipitation was washed 5 times with the volumes of cold methanol and gently mixed. The precipitation was centrifuged at 12,000× *g* for 10 min at 4 °C again to collect the precipitation and repeat once. Then, the methanol was replaced by acetone, and the wash step was repeated twice to remove methanol contamination. Furthermore, the samples were centrifuged at 12,000× *g* for 10 min at 4 °C to collect the precipitation, and the precipitation was dried at room temperature for 5 min. Finally, the samples were centrifuged at 12,000× *g* for 10 min to collect the supernatants. The supernatants were centrifuged again to remove the precipitation completely. Protein concentration was determined by BCA assay and aliquoted to store at −80 °C. Afterwards, 200 μg of protein extract was mixed with a reducing buffer [10 mM DL-dithiothreitol, 8 M urea, 100 mM borane-triethylamine complex (TEAB), pH 8.0], and incubated for 1 h at 60 °C, followed by the addition of iodoacetamide to a final concentration of 50 mM for 10 min in the dark at room temperature (18–22 °C). The filter units were centrifuged at 12,000× *g* for 20 min, and the flow-through was discarded from the collection tube. Then, 100 μL of 100 mM TEAB was added, and the samples were centrifuged at 12,000× *g* for 20 min. This step was repeated three times. The filter units were transferred to new collection tubes, and 100 μL of 100 mM TEAB and 2 μL of sequencing-grade trypsin (1 μg/μL) were added to the samples, which were incubated at 37 °C for 12 h. The samples were centrifuged at 12,000× *g* for 20 min, and the peptide was collected. Then, 50 μL of 100 mM TEAB was added, and the tube was centrifuged again. The collected solution was mixed again, and the solution was lyophilized. The lyophilized samples were resuspended in 50 μL 100 mM TEAB, and 40 μL of each sample were transferred into new tubes for labeling. A total of 88 microliters of acetonitrile was added to the TMT reagent vial at room temperature. The centrifuged reagents were dissolved for 5 min and mixed for centrifugation, and this step was repeated once. Then, 41 μL of the TMT label reagent was added to each sample for mixing. The tubes were incubated at room temperature for 1 h. Finally, 8 μL of 5% hydroxylamine were added to each sample and incubated for 15 min to terminate the reaction. The labeling peptide solutions were lyophilized and stored at −80 °C. (2) For reverse-phase high-performance liquid chromatography analysis, the tryptic peptides were fractionated by high-pH reverse-phase high-performance liquid chromatography using an Agilent Zorbax Extend-C18 column (2.1 × 150 mm, C18, 5 μm, 120 Å, ChromXP Eksigent, Santa Clara, CA, USA). Mobile phases A (2% acetonitrile in HLCP water) and B (98% acetonitrile in HPLC water) were used for the reverse-phase gradient. Briefly, peptides were eluted at a flow velocity of 300 μL/min. The gradient elution conditions were as follows: 0–8 min, 98% A; 8–8.01 min, 98–95% A; 8.01–38 min, 95–75% A; 38–50 min, 75–60% A; 50–50.01 min, 60–10% A; 50.01–60 min, 10% A; 60–60.01 min, 10–98% A; and 60.01–65 min, 98% A. Samples were collected over a period of 8–50 min, and the eluate was collected into the centrifuge tube at one-minute intervals. After the collection and freeze-drying procedures, the samples were frozen and stored at −80 °C for mass spectrometry analysis. Finally, all analyses were performed by a Q-Exactive mass spectrometer (Thermo, Fisher Scientific, Waltham, MA, USA) equipped with a Nanospray Flex source (Thermo, Fisher Scientific, Waltham, MA, USA). Samples were loaded by a capillary C18 trap column (2 cm × 100 µm) and then separated by a C18 column (50 cm × 75 µm) on an EASYnLC 1200 system (Thermo, Fisher Scientific, Waltham, MA, USA). The flow rate was 300 nL/min, and the linear gradient was set as follows: 0~50 min, 2–28% B; 50~60 min, 28–42% B; 60~65 min, 42–90% B; 65~75 min, 90% B. Mobile phase A = 99.9% H_2_O/0.1% formic acid and B = 80% acetonitrile/19.9%H_2_O/0.1% formic acid. Full MS scans were acquired in the mass range of 300–1500 *m*/*z* with a mass resolution of 60,000. The twelve most intense peaks in MS were fragmented with higher-energy collisional dissociation with a collision energy of 32. The MS/MS spectra were obtained with a resolution of 45,000 with a max injection time of 80 ms. The Q-E dynamic exclusion was set for 30.0 s. (3) For bioinformatics analysis, the identified differentially expressed proteins were annotated with a common functional database (http://geneontology.org/, accessed on 18 November 2021).

### 4.7. Statistical Analysis

Data were analyzed using one-way analysis of variance and compared with the significant difference multiple comparison test using SPSS version 25 (Armonk, NY, USA). Differences were considered significant at *p* ≤ 0.05. Figures were created using SigmaPlot (version 12.5).

## 5. Conclusions

Accordingly, we suggest that PET is mainly affected by the abundance of PS II, OEC, cytb_6_/f, P_C_, and PS I in *Hosta* ‘Sum and Substance’ and ‘Fire Island’ grown under weak light; the improved photosynthetic capacity of both the *Hosta* varieties under weak growth light was mainly related to the enhancement of light energy absorption and capture of PS II, as well as an increase in the abundance of Rubisco.

## Figures and Tables

**Table 1:** Changes in the protein abundance of *Hosta* ‘Sum and Substance’ and ‘Fire Island’ grown under 50% (50%FL) and 10% full sunlight (10%FL). Changes in the protein expression levels are shown as fold change (↑ and ↓) of the 10%FL relative to the 50%FL. The large changes of protein expression are indicated in ↑ (elevated) and ↓ (decreased). The names of the proteins that were identified are listed and classified by function. The difference was statistically significant between light treatments (*p* < 0.05 level) (*p* < 0.05).

Protein Identification		Protein Name	Fold Change	
Protein Types	Protein IDs		‘Sum and Substance’	‘Fire Island’
Rubisco	CL2106.Contig5_All	Rubisco large subunit		2.16 ↑
	CL4327_Contig1_All	Rubisco large subunit		2.03 ↑
	Unigene52506_All	Rubisco small subunit	3.44 ↑	
PS II	CL24649_Contig5_All	PS II protein D1		4.8 ↑
	CL12408_Contig5_All	PS II 47KDa protein		2.04 ↑
	Unigene121480_All	PS II protein D1		4.06 ↑
	CL6272_Contig6_All	PS II	3.49 ↑	
OEC	CL11213_Contig1_All	PS II oxygen-evolving complex		−2.56 ↓
	CL9703_Contig3_All	PS II oxygen-evolving complex		−2.61 ↓
	CL11958_Contig10_All	PS II oxygen-evolving complex	−2.81 ↓	
	CL30452_Contig2_All	PS II oxygen-evolving complex	−3.32 ↓	
	Unigene45629_All	PS II oxygen-evolving complex	−3.54 ↓	
ETC	CL26278_Contig7_All	Cytochrome b6-f complex	−1.37 ↓	
	CL26278_Contig3_All	Cytochrome b6-f complex	−1.31 ↓	−2.4 ↓
	Unigene12003_All	Cytochrome b6-f complex		−2.91 ↓
	CL7316_Contig4_All	Plastocyanin 1		−11.81 ↓
PS I	CL1094_Contig1_All	PS I reaction center subunit VI		−2.19 ↓
	CL11407_Contig2_All	PS I reaction center subunit N		−3.05 ↓
	Unigene725_All	PS I reaction center subunit III		−4.94 ↓
	CL2739_Contig2_All	Photosynthetic electron transport in PS I		−2.83 ↓
	CL26881_Contig1_All	Photosynthetic electron transport in PS I	−2.14 ↓	
PSI antenna proteins	Unigene124018_All	PS I chlorophyll a/b-binding protein 5	−3.17 ↓	−2.13 ↓
	Unigene9117_All	PS I chlorophyll a/b-binding protein 4	−2.53 ↓	−3.86 ↓
	CL29551_Contig2_All	PS I chlorophyll a/b-binding protein		−2.41 ↓
	CL31036_Contig7_All	PS I chlorophyll a/b-binding protein 1		−61.06 ↓
	CL31202_Contig6_All	PS I chlorophyll a/b-binding protein 3		−5.33 ↓
	CL32131_Contig2_All	PS I chlorophyll a/b-binding protein 4		−5.07 ↓
	Unigene12354_All	PS I chlorophyll a/b-binding protein 2		−5.11 ↓
NPQ	Unigene435_All	Chlorophyll a/b-binding protein CP24 10B		−2.48 ↓
	CL25573_Contig1_All	Chlorophyll a/b-binding protein CP24 10B		−7.87 ↓
	CL28869_Contig9_All	photosystem II 22 kDa protein		−2.02 ↓

## Data Availability

The original contributions presented in the study are included in the article, further inquiries can be directed to the corresponding author.
